# Psychometric validation of the Japanese version of Alcohol Quality of Life Scale (AQoLS-Japan) in the treatment of patients with alcohol use disorder

**DOI:** 10.1007/s11136-019-02310-w

**Published:** 2019-10-04

**Authors:** Susumu Higuchi, Yoshiya Moriguchi, Kristin Hui Xian Tan

**Affiliations:** 1 National Hospital Organization Kurihama Medical and Addiction Center, 5-3-1 Nobi, Yokosuka, Kanagawa 239-0841 Japan; 2 Lundbeck Japan K.K, Tokyo, Japan; 3 Lundbeck Singapore Pte Ltd, Singapore, Singapore

**Keywords:** Alcohol use disorders, Quality of life, Japan

## Abstract

**Purpose:**

The Alcohol Quality of Life Scale (AQoLS) is accepted as a useful measure in assessing impact of alcohol use disorders (AUD) on health-related quality of life (HR-QoL) in Western cultures. We aimed to assess the psychometric properties of the Japanese version of the AQoLS (AQoLS-Japan).

**Methods:**

This was a 3-month, observational cohort study in patients undergoing routine treatment for AUD in Japan. HR-QoL was assessed using the AQoLS-Japan (34 items, 7 dimensions). Scale psychometrics were analyzed using correlative techniques.

**Results:**

Data from 132 patients were analyzed. Inter-item and item-scale correlations for the AQoLS-Japan scale were moderate to strong. Confirmatory factor analysis results supported the AQoLS-Japan structure but there was evidence of interdependency among some items and factors. Cronbach’s alpha coefficients for internal consistency ranged from 0.73 to 0.97, and intraclass correlation coefficients for scores between test (baseline) and retest (2 weeks) ranged from 0.65 to 0.82. Convergent and divergent validity and known-groups validity were supported. Evaluation of within-group change demonstrated that the AQoLS-Japan total and domains consistently demonstrated statistically significant improvement (*p* < 0.001 in all cases) in HR-QoL over time. Estimates for minimal clinically important difference on the AQoLS-Japan total score ranged from 13.2 to 18.2 for group-level change and from 2.4 to 15.7 for a group-level difference.

**Conclusions:**

The AQoLS-Japan is a reliable and valid measure of HR-QoL that is able to demonstrate benefits associated with the routine treatment of AUD in Japan.

**Electronic supplementary material:**

The online version of this article (10.1007/s11136-019-02310-w) contains supplementary material, which is available to authorized users.

## Introduction

Alcohol use disorders (AUD), including alcohol abuse and dependence, pose a great health, social and economic burden to affected individuals, their families, and society. The harmful use of alcohol is one of the world’s leading health risks [1], and the greater the volume of alcohol that an individual consumes, the greater the risks associated with its use. In 2018, the World Health Organization (WHO) estimated that 22.8% of the general population (aged 15 + years) participate in heavy episodic drinking, and that 3.4% of the Japanese population have an AUD (12-month prevalence estimate) [2]. However, various surveys have reported that the majority (up to 70%) of Japanese heavy drinkers are unaware that their drinking behavior may be problematic [3] and only about 5% of alcohol-dependent persons in Japan actually seek medical advice and treatment [4]. It has been suggested that this lack of awareness is, at least partly, attributable to Japanese drinking culture, where there is high tolerance for drinking alcohol to facilitate socialization [5]. Conversely, there is significant stigma associated with alcoholism in Japan, where people with AUDs are often stereotyped as having ‘weak will’ and socially unacceptable which likely deters them from seeking treatment [6].

In order to be able to assess the effectiveness of potential alcohol reduction strategies in a ‘people first’ approach, the field has started to move away from traditional measures of drinking (e.g., total alcohol consumption [TAC], or number of heavy drinking days [HDD]) to additional outcomes that are closer to patient needs. Health-related quality of life (HR-QoL) is acknowledged as critical domain to consider when assessing the effectiveness of treatment for AUDs and is often included as a secondary endpoint in clinical trials [7, 8]. However, a 2012 systematic review of AUD studies using generic HR-QoL scales found that they were not well suited to showing treatment effects in subjects with AUD [9]. Thus, the Alcohol Quality of Life Scale (AQoLS) was developed as the first AUD-specific measure of HR-QoL for increased sensitivity in showing the effectiveness of therapeutic interventions from the patient’s perspective [10].

Scale psychometrics of the French and English (UK) versions of the AQoLS have been evaluated and have demonstrated construct validity and internal consistency [11]. The development process for the original scale also included a formal translatability assessment by translation experts to ensure the items were amenable to translation and cultural adaptation. The original English (UK) version of AQoLS was translated into Japanese, and formal cultural adaptation was completed in July 2013 [9]. We present here the first assessment of the psychometric properties of the Japanese version of the Alcohol Quality of Life Scale (AQoLS-Japan) in Japanese patients with an AUD.

## Methods

This was a multi-site, observational, prospective, longitudinal, cohort study in patients with AUD, followed in specialized care for up to 3 months (± 2 weeks) in Japan. Patients were treated according to routine practice, i.e., treatment was not decided in advance by the study protocol. The study was conducted between October 4, 2016 and September 5, 2017, at 15 outpatient sites across Japan.

### Patients

Patients were male and female Japanese adults (aged ≥ 20 years) with a diagnosis of AUD according to the *Diagnostic and Statistical Manual of Mental Disorders, 5th Edition* (*DSM*-*5*). Eligible patients had to be currently on an established outpatient treatment plan with the intent to follow that plan for 3 months or have an established treatment plan that started within 1–4 weeks after the baseline visit. Key exclusion criteria were any serious or unstable psychiatric disorders (such as drug addiction) and physical disorders that prevented participation into the study, learning difficulties that prevented him/her reading and understanding questionnaires (e.g., dementia), and, in the physician’s opinion, could not be followed for the whole duration of the study.

### Assessments

Assessments were conducted as part of routine practice visits at baseline, 2 weeks (± 1 week), and 3 months (± 2 weeks). Patients self-rated their HR-QoL using the AQoLS-Japan which has 34 items across 7 dimensions: activities (items 2–7, 13, 15, and 25–26), relationships (items 1, 8–11, and 27), living conditions (items 16–18 and 24), negative emotions (items 22–23), control (items 28–32), sleep (items 33–34), and self-esteem (items 12, 14, and 19–21) (Supplementary Appendix Table e1). Each item has four response categories (not at all, a little, quite a lot, and very much), with a 4-week recall period. Dimension and total (sum of 34 items) scores are linearly transformed to a 0–100 range, with higher scores indicating poorer HR-QoL. Other HR-QoL assessments included Japanese versions of generic HR-QoL measures—the EuroQol questionnaire (EQ-5D-3L) [12] and the SF-36 Health Survey (version 2) (SF-36) [13]. Clinicians and patients rated their global impressions of severity using the 7-item Clinical Global Impression of Severity (CGI-S) and the 5-item Patient Global Impression of Severity (PGI-S), respectively. Impressions of change were assessed using the Clinical Global Impression of Improvement (CGI-I) and Patient Global Impression of Change (PGI-C) [14], both consisting of 7 items where 1 = very much improved/better, 2 = much improved/better, 3 = minimally improved/better, 4 = no change, 5 = minimally worse, 6 = much worse, and 7 = very much worse). Levels of alcohol consumption were evaluated using the Timeline Follow-Back method (past 28 days) [15]. Drinking risk levels (DRLs) were defined according to WHO criteria as [male/female] low ≤ 40 g/≤ 20 g; medium 41–60 g/21–40 g; high 61–100 g/41–60 g; and very high > 100 g/> 60 g per day) [2]. A DRL response was defined as from very high to medium/low DRL, high to low DRL, medium to low DRL, or low DRL to alcohol consumption of 0 g/day.

### Statistical analyses

#### Sample size estimation

The target sample size was determined based on the requirement to have approximately 60 patients for the assessment of test–retest reliability and responsiveness [16]. Based on the results of two previous clinical trials in patients with AUD [17, 18], it was estimated that approximately 59% of patients would maintain their baseline DRL after 2 weeks of follow-up and 58% would achieve treatment response at 3 months. Hence, considering 18% withdrawal rate at 3 months, it was estimated that a minimum of 127 patients were needed for this study. To allow for any cultural differences between Japan and the countries involved in the clinical trials (e.g., higher loss to follow-up), the minimum enrollment was increased to 150 patients.

#### Analysis population

The analysis population included all patients who met selection criteria and completed the baseline assessment. Psychometric validation assessments were performed for all patients who completed all study visits.

#### Descriptive statistics

Standard descriptive statistics were used to describe the distributional properties of the AQoLS-Japan item, dimension, and total scores at each study visit, as well as for change from baseline for AQoLS-Japan dimension and total scores at follow-up visits.

Descriptive statistics were also used to summarize the HR-QoL and clinical status of patients with AUD (AQoLS-Japan, EQ-5D, SF-36, PGI-S, and PGI-C) at baseline and 3 months.

#### Psychometric validation

Dimensional structure was assessed through inter-item and item-scale correlations at each visit and confirmatory factor analysis (CFA) using data from baseline. Adequacy of model fit was evaluated through the model Chi-square test statistic, comparative fit index (CFI ≥ 0.95), Tucker–Lewis index (TLI ≥ 0.95), and the root mean square error of approximation (RMSEA ≤ 0.06). Internal reliability of AQoLS-Japan was evaluated through Cronbach’s alpha coefficients, where an alpha between 0.70 and 0.90 indicates a set of items that is strongly related but not redundant and that is capable of supporting a unidimensional scoring structure [19].

Test–retest reliability was evaluated in patients whose condition remained stable on PGI-C, PGI-S, CGI-I, and DRL between baseline (test) and 2 weeks (retest) by estimating the intraclass correlation coefficients (ICCs) for scores between test and retest; ICCs of ≥ 0.70 were taken to represent adequate reliability. Construct validity was investigated by testing a priori hypotheses to evaluate the direction and strength of the relationships between AQoLS-Japan scores and scores on comparator measures (SF-36, EQ-5D, PGI-S, CGI-S, and alcohol consumption) using Pearson product-moment correlations at each visit. The strength of correlations is assessed based on Cohen’s criteria [20]; correlations between 0.10 and 0.29 are considered small, correlations between 0.30 and 0.49 are considered moderate, and correlations of 0.50 or greater are considered strong. A priori hypotheses regarding the direction and strength of these correlations were determined based on the literature and findings from the UK and French AQoLS psychometric validation [8, 11]. We hypothesized that there would be:

1. Moderate to strong negative correlation between the AQoLS and the SF-36 mental and role-social components.
2. Moderate negative correlations between the AQoLS and the SF-36 role emotional, vitality, mental health, and social functioning components.
3. Small to moderate negative correlation between the AQoLS and the EQ-5D visual analogue scale.
4. Low to moderate positive correlation between the AQoLS and the CGI-S; stronger correlation between the AQoLS and the PGI-S.
5. Low to moderate positive correlations between the AQoLS and measures of alcohol consumption.

Since there is a well-established, ‘dose-related’ continuum of health impact for AUD [21], we wanted to check scale validity across the spectrum of drinking behaviors, from mild through to more severe disease and at different levels of alcohol consumption. Known-groups validity was evaluated through statistical significance of differences in AQoLS-Japan scores between the two most extreme subgroups across known prespecified subgroups (disease severity based on PGI-S and CGI-S, and level of consumption based on number of HDDs, number of drinking days and DRL) using t tests at each visit.

The ability to detect change of the AQoLS-Japan was evaluated by using Pearson product-moment correlation coefficients between AQoLS-Japan Month-3 change from baseline and Month-3 change on comparator measures (SF-36, EQ-5D, alcohol consumption, PGI-C, and CGI-I). AQoLS-Japan change in scores between baseline and 3 months were also computed for patients who improved on PGI-C, CGI-I, and reduced alcohol consumption during the 3-month follow-up period and the significance of change was tested using paired *t* test. In addition, effect-size estimate of change and standardized response mean were computed for the mean change in scores between baseline and 3 months on the AQoLS dimensions and total. Responsiveness effect sizes were interpreted based on [22] guidelines [22], where ≥ 0.20 to < 0.50 represent small effects, ≥ 0.50 to < 0.80 represent moderate effects, and ≥ 0.80 represent large effects. The minimal clinically important difference (MCID) is defined as the smallest change or difference in scores of a measure perceived by patients as beneficial or harmful [23]. Estimates for an MCID to evaluate group-level change over time on the AQoLS total were determined using the AQoLS total mean change for patients who had a small improvement (defined as PGI-S = 1, PGI-C = 3, CGI-S = 1 or 2, CGI-I = 3, or DRL improvement of one category) between baseline and Month 3 on each anchor determined to be adequate [23–25]. Estimates for responder definitions were based on mean changes, receiver-operator characteristic (ROC) analysis, and cumulative distribution function plots.

All statistical tests were two-sided and conducted at the 5% level of significance. With the exception of the SF-36 (where missing data at the item level were treated in accordance with standard scoring guidelines [13]), imputation was not performed for missing data. The statistical software used was SAS^®^, Version 9.4.

## Results

### Patient disposition and baseline characteristics

A total sample of 150 patients completed baseline assessments and were included in the study. Of these, five patients were lost to follow-up between baseline and the Week-2 visit and a further 12 were lost to follow-up between Week 2 and Month 3. Data from 132 of the 133 patients who completed all study visits were used for psychometric validation as one patient did not complete the AQoLS-Japan at Month-3 visit.

Most patients (82.0%) were male and the average age was 52.9 years. Baseline demographic and clinical characteristics for patients are shown in Table 1 and were similar between the total sample of 150 patients and the 132 patients with AQoLS-Japan data considered for psychometric validation. The most common therapeutic goal was to abstain completely from alcohol (89.9%). Most enrolled patients (60.0%) were continuing on their current treatment plan (average of 4.7 years since initiation of treatment plan), with the rest initiating their plan at the start of the study. In line with routine care in Japan, all patients received some form of non-pharmacologic therapy (brief advice 41.1%, individual therapy 93.0%, group therapy 43.4%, education program 41.1%, and family intervention 13.2%). Over half (58.9%) of patients received pharmacologic treatment at baseline (38.8% received acamprosate and 13.2% received disulfiram).

**Table 1:** Baseline characteristics

	Overall (*N* = 150)	Psychometric analysis group (*N* = 132)
Sex; *n* (%)		
Male	123 (82.0%)	108 (81.8%)
Female	27 (18.0%)	24 (18.2%)
Age (years); mean (SD)	52.9 (12.1)	53.2 (12.4)
Living status; *n* (%)		
Alone	58 (38.7%)	51 (38.6%)
Not alone	92 (61.3%)	81 (61.4%)
Employment status; *n* (%)		
On a job	62 (41.3%)	51 (38.6%)
Working at home	6 (4.0%)	6 (4.5%)
Retired	18 (12.0%)	17 (12.9%)
No job	64 (42.7%)	58 (43.9%)
Marital status; *n* (%)		
Not married	36 (24.0%)	32 (24.2%)
Married/living with someone	65 (43.3%)	54 (40.9%)
Divorced/separated	45 (30.0%)	42 (31.8%)
Bereavement	4 (2.7%)	4 (3.0%)
Current smoker; *n* (%)	100 (67.1%)	90 (68.7%)
Alcohol use disorder; *n* (%)		
Abuse	4 (2.7%)	3 (2.3%)
Dependence	146 (97.3%)	129 (97.7%)
Total alcohol consumption (g/day) over 28 days; mean (SD)	37.1 (48.1)	33.3 (43.9)
Number of drinking days; mean (SD)	11.8 (11.1)	10.8 (10.9)
Number of heavy drinking days; mean (SD)	8.5 (10.4)	7.4 (9.8)
Age at onset of drinking problem (years); mean (SD)	39.0 (13.7)	38.9 (13.7)
Time since diagnosis (years); mean (SD)	3.8 (5.4)	4.1 (5.5)
Number of previous attempts to abstain or reduce alcohol consumption; mean (SD)	9.0 (20.1)	9.8 (21.3)
Comorbidities; *n* (%)		
Cardiovascular	33 (22.0%)	30 (22.7%)
Gastric	28 (18.7%)	23 (17.4%)
Hepatic	64 (42.7%)	54 (40.9%)
Metabolic	27 (18.0%)	22 (16.7%)
Psychiatric	55 (36.7%)	48 (36.4%)

### Treatment effect

Reductions in alcohol consumption with routine treatment were observed at 2 weeks and 3 months. Mean TAC was reduced from a mean of 37.1 g/day at baseline to 15.0 g/day at 2 weeks and to 11.8 g/day at 3 months. The number of drinking days reduced from a mean of 11.8 drinking days (42.0% of days drinking) at baseline to 4.0 drinking days (14.3% of days drinking) at 3 months. Overall 57.1% (56/98) patients showed a DRL response by 3 months.

Reductions in drinking measures were reflected in patient and clinician impressions of change and in HR-QoL. According to the PGI-C, 71.1% of study patients improved at 2 weeks and 80.2% improved at 3 months. According to CGI-I, 58.4% of study patients improved at 2 weeks and 58.6% improved at 3 months. The mean ± SD AQoLS-Japan total score was 30.4 ± 22.5 at baseline reducing to 16.8 ± 18.4 at 3 months, indicating an improvement (Table 2). Patients also showed improvement in generic measures of HR-QoL. Mean EQ-5D visual analogue scale scores improved from 68.5 at baseline to 75.3 at 3 months. In terms of the SF-36, the largest improvements were seen in the dimension ‘role limitations due to emotional problems’ (change in mean scores of 6.1), followed by ‘vitality’ (5.7), and ‘mental health’ (5.1) (Supplementary Table e3).

**Table 2:** AQoLS-Japan dimension and total descriptive statistics: baseline, Week-2, and Month-3 visits

AQoLS dimension	Baseline	Week 2	Month 3
Total			
*n*	144	135	129
Mean (SD)	30.4 (22.5)	26.6 (22.2)	16.8 (18.4)
Median (Q1, Q3)	26.5 (10.3, 45.6)	22.5 (5.9, 40.2)	9.8 (2.0, 24.5)
Minimum, maximum	0.0, 83.3	0.0, 91.2	0.0, 83.3
*n* (%) scoring scale minimum	6 (4.0)	5 (3.4)	14 (10.6)
*n* (%) scoring scale maximum	0 (0.0)	0 (0.0)	0 (0.0)
*n* (%) missing	6 (4.0)	10 (6.9)	3 (2.3)
Activities			
*n*	147	139	130
Mean (SD)	32.4 (25.5)	26.7 (24.4)	16.3 (19.6)
Median (Q1, Q3)	26.7 (10.0, 53.3)	20.0 (3.3, 43.3)	10.0 (0.0, 20.0)
Minimum, maximum	0.0, 96.7	0.0, 96.7	0.0, 86.7
*n* (%) scoring scale minimum	19 (12.7)	23 (15.9)	33 (25.0)
*n* (%) scoring scale maximum	0 (0.0)	0 (0.0)	0 (0.0)
*n* (%) missing	3 (2.0)	6 (4.1)	2 (1.5)
Relationships			
*n*	148	142	132
Mean (SD)	25.4 (22.6)	22.7 (23.0)	15.5 (20.3)
Median (Q1, Q3)	22.2 (5.6, 38.9)	16.7 (5.6, 33.3)	5.6 (0.0, 27.8)
Minimum, maximum	0.0, 83.3	0.0, 94.4	0.0, 94.4
*n* (%) scoring scale minimum	26 (17.3)	34 (23.4)	60 (45.5)
*n* (%) scoring scale maximum	0 (0.0)	0 (0.0)	0 (0.0)
*n* (%) missing	2 (1.3)	3 (2.1)	0 (0.0)
Living conditions			
*n*	148	144	132
Mean (SD)	21.4 (22.6)	16.7 (19.7)	11.2 (17.2)
Median (Q1, Q3)	16.7 (0.0, 33.3)	8.3 (0.0, 25.0)	0.0 (0.0, 16.7)
Minimum, maximum	0.0, 91.7	0.0, 83.3	0.0, 66.7
*n* (%) scoring scale minimum	43 (28.7)	56 (38.6)	75 (56.8)
*n* (%) scoring scale maximum	0 (0.0)	0 (0.0)	0 (0.0)
*n* (%) missing	2 (1.3)	1 (0.7)	0 (0.0)
Negative emotions			
*n*	148	145	132
Mean (SD)	44.9 (31.0)	42.8 (30.7)	27.7 (29.3)
Median (Q1, Q3)	33.3 (33.3, 66.7)	50.0 (16.7, 66.7)	16.7 (0.0, 50.0)
Minimum, maximum	0.0, 100.0	0.0, 100.0	0.0, 100.0
*n* (%) scoring scale minimum	27 (18.0)	29 (20.0)	54 (40.9)
*n* (%) scoring scale maximum	13 (8.7)	9 (6.2)	4 (3.0)
*n* (%) missing	2 (1.3)	0 (0.0)	0 (0.0)
Control			
*n*	150	144	131
Mean (SD)	31.6 (29.2)	26.0 (28.9)	13.8 (22.2)
Median (Q1, Q3)	26.7 (0.0, 53.3)	16.7 (0.0, 40.0)	0.0 (0.0, 20.0)
Minimum, maximum	0.0, 100.0	0.0, 100.0	0.0, 100.0
*n* (%) scoring scale minimum	42 (28.0)	48 (33.1)	75 (56.8)
*n* (%) scoring scale maximum	5 (3.3)	3 (2.1)	1 (0.8)
*n* (%) missing	0 (0.0)	1 (0.7)	1 (0.8)
Sleep			
*n*	149	145	132
Mean (SD)	39.3 (33.3)	35.3 (33.4)	25.1 (29.9)
Median (Q1, Q3)	33.3 (0.0, 66.7)	33.3 (0.0, 66.7)	16.7 (0.0, 33.3)
Minimum, maximum	0.0, 100.0	0.0, 100.0	0.0, 100.0
*n* (%) scoring scale minimum	42 (28.0)	43 (29.7)	60 (45.5)
*n* (%) scoring scale maximum	17 (11.3)	16 (11.0)	9 (6.8)
*n* (%) missing	1 (0.7)	0 (0.0)	0 (0.0)
Self-esteem			
*n*	149	143	132
Mean (SD)	32.6 (28.2)	29.2 (26.4)	22.1 (25.3)
Median (Q1, Q3)	26.7 (6.7, 53.3)	26.7 (6.7, 46.7)	13.3 (0.0, 33.3)
Minimum, maximum	0.0, 100.0	0.0, 100.0	0.0, 100.0
*n* (%) scoring scale minimum	23 (15.3)	32 (22.1)	46 (34.8)
*n* (%) scoring scale maximum	4 (2.7)	1 (0.7)	1 (0.8)
*n* (%) missing	1 (0.7)	2 (1.4)	0 (0.0)

### Psychometric validation of the AQoLS-Japan

#### Distributional properties

Mean baseline dimension and total AQoLS-Japan scores were < 50 on the 0-to-100 scale, reflecting the mild severity of disease in the sample (Table 2). Mean scores were slightly reduced with routine treatment (indicating improvement) between baseline and Week 2 and subsequently reduced further at the Month-3 visit (Table 2). Floor effects (> 20% of the sample scoring at the minimum) were evident for the living conditions, control, and sleep dimensions, with the most substantial floor effects for the individual item of ‘housing situation.’ Ceiling effects were largely absent at all time points, and the AQoLS-Japan total score had no or only minimal floor or ceiling effects at any time point.

#### Dimensional structure

Inter-item and item-scale correlations at baseline, Week 2, and Month 3 were moderate to strong. Very strong correlations (> 0.8), suggesting potential redundancy, were found among items in the activities (items 2–7, 13, 15, and 25–26), control (items 28–32), and sleep (items 33 and 34) dimensions. The potential for redundancy among individual items in these domains was further indicated by the high corrected item-scale correlations (> 0.8) for a number of these items. Factor loadings from the CFA were all positive and statistically significant (*p* < 0.05). Two of the goodness-of-fit indices supported the adequacy of the AQoLS-Japan measurement model but one (the RMSEA) was higher than the recommended maximum value. Thus, although the CFA results generally supported the 7-factor structure of the AQoLS-Japan, there was evidence of interdependency among some items and factors (Supplementary Table e4).

#### Reliability

Cronbach’s alpha coefficients for internal consistency at baseline ranged from 0.73 (living condition) to 0.97 (total score) (Table 3). The highest ICCs for test–retest reliability were found for the PGI-C stable subsample which was the primary indicator of stability in this evaluation. The total score and all dimensions except living conditions and sleep had reliability coefficients above the minimum recommended threshold of 0.70. However, the lower bound of the 95% confidence interval fell below this threshold for all scores except the total, relationships, and self-esteem.

**Table 3:** Reliability of the AQoLS-Japan

AQoLS-Japan dimension	AQoLS-Japan Cronbach’s alpha coefficients for internal consistency			Intraclass correlation coefficients for test–retest reliability over 2 weeks (95% CI)
	Baseline	Week 2	Month 3	
Total	0.97	0.97	0.97	0.80 (0.70, 0.88)
Activities	0.92	0.93	0.91	0.74 (0.60, 0.83)
Relationships	0.83	0.87	0.86	0.80 (0.70, 0.87)
Living conditions	0.73	0.72	0.75	0.65 (0.50, 0.77)
Negative emotions	0.83	0.82	0.81	0.74 (0.62, 0.83)
Control	0.92	0.94	0.93	0.70 (0.56, 0.80)
Sleep	0.93	0.93	0.94	0.65 (0.49, 0.76)
Self-esteem	0.91	0.91	0.92	0.82 (0.72, 0.88)

Intraclass correlation coefficients are shown for the stable subsample (based on PGI-C, *n* = 74)

#### Construct validity

**Fig. 1:**
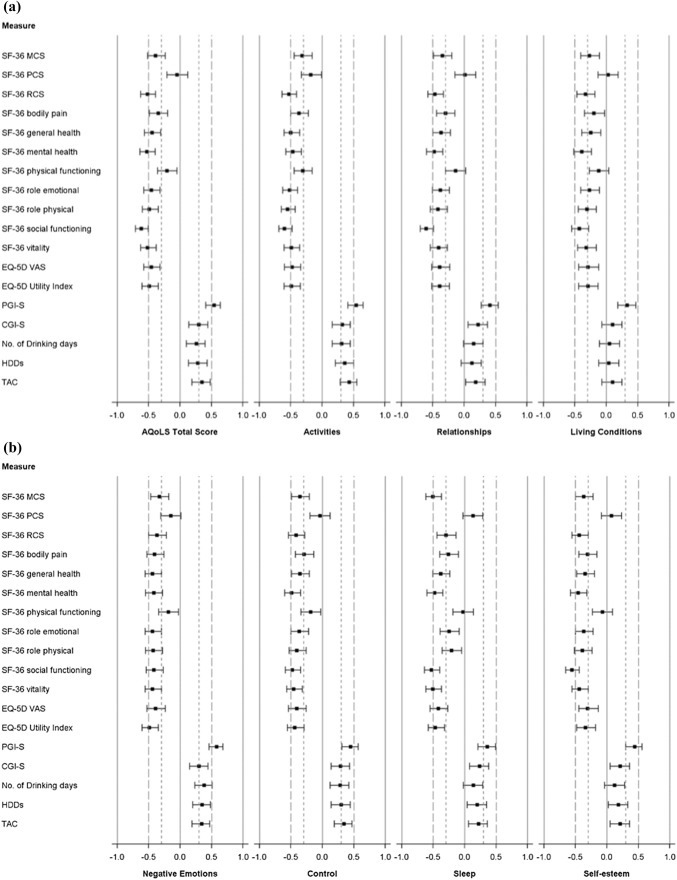
Construct validity: correlations between scores on the AQoLS and other measures at baseline. **a** AQoLS Total, activities, relationships, and living conditions dimensions. **b** AQoLS negative emotions, control, sleep, and self-esteem dimensions. *AQoLS* Alcohol Quality of Life Scale, *CGI-S* clinical global impression–severity, *HDD* heavy drinking day, *MCS* mental component summary, *PCS* physical component summary, *PGI-S* patient global impression–severity, *TAC* total alcohol consumption

Convergent and divergent correlations (Fig. 1) confirmed moderate to strong negative (*|r| *≥ 0.30) correlations with the SF-36 mental and role component summaries, but correlations were negligible to small for the physical component (*|r| *< 0.30; [20]), and small to moderate for the EQ-5D visual analogue scale (*|r| *= 0.29–0.59). Small to moderate positive correlations were found with the CGI-S (*|r|* = 0.10–0.39) and alcohol consumption measures (*|r| *= 0.04–0.55), with higher positive correlations with the PGI-S (*|r| *= 0.28–0.58). Differences across known groups were statistically significant (*p* < 0.05) for most comparisons with PGI-S and CGI-S at baseline, with the only non-significant exception being the association between CGI-S and living conditions. There was also a clear pattern of higher scores (indicating worse HR-QoL) with increasing levels of drinking-days consumption across most of the AQoLS-Japan dimensions. Baseline differences between the low (< 5 HDDs) and high HDD (≥ 14 HDDs) groups were statistically significant (*p* < 0.05) for most AQoLS-Japan dimensions except relationships and living conditions. Similarly, statistically significant (*p* < 0.05) baseline differences were observed between patients with low and high or very high DRL on all AQoLS dimensions except living conditions.

#### Responsiveness

In general, the pattern of associations with the SF-36 and consumption measures was similar to that observed with the cross-sectional scores in the convergent and divergent construct validity assessments, with moderate correlations between AQoLS-Japan change scores and changes on the mental domains of the SF-36 but weaker correlations with changes on the physical domains of the SF-36. Changes in AQoLS-Japan scores were generally more highly associated with change on PGI-C than CGI-I. Evaluation of within-group change demonstrated that the AQoLS-Japan total and all domains were consistently able to demonstrate statistically significant improvement (*p* < 0.001 in all cases) in HR-QoL over time. Mean ± SD change on the AQoLS-Japan total was − 13.7 ± 20.9 for the overall sample, which increased to between − 16.7 ± 21.7 and − 24.9 ± 18.9 for patients who were classified as having improved on the global impression of change assessments and alcohol consumption measures, respectively. Estimates for effect size and standardized response means for the AQoLS total and dimension scores were small to moderate (i.e., ≥ 0.20 to < 0.80) for change in the overall sample and moderate (i.e., ≥ 0.50 to < 0.80) for change associated with a defined improvement based on PGI-C and CGI-I. For most of the AQoLS dimensions (except for sleep and self-esteem), the estimates for effect size and standardized response means were large (i.e., ≥ 0.80) for change associated with a defined improvement as measured by HDDs and TAC (Table 4).

**Table 4:** Ability to detect change—effect-size estimates and standardized response means

AQoLS dimension	Change in AQoLS Mean (SD), *n*	t-Statistic (*p* value)	Effect-size estimate	Standardized response mean
Whole sample				
Total score	− 13.7 (20.9), 124	7.33 (< 0.001)	− 0.61	− 0.66
Activities	− 15.9 (24.7), 127	7.23 (< 0.001)	− 0.62	− 0.64
Relationships	− 10.1 (22.4), 131	5.17 (< 0.001)	− 0.45	− 0.45
Living conditions	− 10.8 (23.8), 130	5.19 (< 0.001)	− 0.48	− 0.45
Negative emotions	− 16.9 (29.5), 130	6.54 (< 0.001)	− 0.55	− 0.57
Control	− 16.7 (28.3), 131	6.78 (< 0.001)	− 0.57	− 0.59
Sleep	− 12.5 (33.7), 131	4.24 (< 0.001)	− 0.37	− 0.37
Self-esteem	− 10.4 (25.3), 131	4.72 (< 0.001)	− 0.37	− 0.41
PGI-C improved (score ≤ 3 at Month 3)				
Total score	− 16.7 (21.7), 99	7.66 (< 0.001)	− 0.72	− 0.77
Activities	− 18.8 (25.7), 102	7.36 (< 0.001)	− 0.74	− 0.73
Relationships	− 13.4 (23.0), 104	5.95 (< 0.001)	− 0.58	− 0.58
Living conditions	− 14.9 (24.0), 103	6.31 (< 0.001)	− 0.61	− 0.62
Negative emotions	− 18.8 (30.6), 103	6.23 (< 0.001)	− 0.59	− 0.61
Control	− 21.6 (28.2), 104	7.80 (< 0.001)	− 0.74	− 0.76
Sleep	− 16.2 (33.0), 104	5.00 (< 0.001)	− 0.49	− 0.49
Self-esteem	− 13.1 (26.5), 104	5.03 (< 0.001)	− 0.45	− 0.49
CGI-I improved (score ≤ 3 at Month 3)				
Total score	− 17.5 (20.2), 75	7.50 (< 0.001)	− 0.79	− 0.87
Activities	− 21.4 (25.9), 76	7.21 (< 0.001)	− 0.84	− 0.83
Relationships	− 13.6 (21.0), 77	5.69 (< 0.001)	− 0.59	− 0.65
Living conditions	− 14.5 (21.2), 76	5.94 (< 0.001)	− 0.70	− 0.68
Negative emotions	− 21.4 (29.0), 77	6.49 (< 0.001)	− 0.69	− 0.74
Control	− 23.2 (27.6), 78	7.40 (< 0.001)	− 0.81	− 0.84
Sleep	− 13.0 (32.5), 77	3.50 (< 0.001)	− 0.38	− 0.40
Self-esteem	− 13.0 (25.5), 77	4.46 (< 0.001)	− 0.47	− 0.51
HDD improved (0–4 HDDs at 3 months, excluding patients who had ≤ 4 HDDs at baseline)				
Total score	− 24.9 (18.9), 37	8.00 (< 0.001)	− 1.55	− 1.31
Activities	− 29.8 (24.7), 39	7.54 (< 0.001)	− 1.52	− 1.21
Relationships	− 17.1 (22.1), 40	4.89 (< 0.001)	− 0.80	− 0.77
Living conditions	− 17.5 (21.3), 39	5.14 (< 0.001)	− 0.90	− 0.82
Negative emotions	− 30.8 (34.3), 39	5.60 (< 0.001)	− 1.28	− 0.90
Control	− 35.4 (23.8), 41	9.53 (< 0.001)	− 1.65	− 1.49
Sleep	− 26.7 (35.8), 40	4.72 (< 0.001)	− 0.79	− 0.75
Self-esteem	− 15.8 (25.4), 40	3.94 (< 0.001)	− 0.61	− 0.62
TAC improved (50% reduction in TAC between baseline and 3 months)				
Total score	− 21.6 (19.2), 58	8.59 (< 0.001)	− 1.20	− 1.13
Activities	− 26.1 (24.3), 60	8.34 (< 0.001)	− 1.24	− 1.08
Relationships	− 15.6 (22.1), 61	5.50 (< 0.001)	− 0.72	− 0.70
Living conditions	− 16.5 (20.5), 60	6.25 (< 0.001)	− 0.85	− 0.81
Negative emotions	− 27.5 (31.0), 60	6.88 (< 0.001)	− 1.07	− 0.89
Control	− 31.1 (27.0), 62	9.05 (< 0.001)	− 1.27	− 1.15
Sleep	− 17.8 (37.9), 61	3.66 (< 0.001)	− 0.52	− 0.47
Self-esteem	− 14.3 (24.6), 61	4.55 (< 0.001)	− 0.53	− 0.58

#### Interpretation of change

Estimates for the MCID of group-level change on AQoLS-Japan total scores were − 18.2 based on PGI-S, − 13.2 based on PGI-C, and − 13.5 based on DRL (Table 5). For group-level differences, the MCID of group-level differences were − 15.7 based on PGI-S, − 9.7 based on PGI-C, and − 2.4 based on DRL.

**Table 5:** Mean scores and correlations for AQoLS Total change from baseline scores at 3 months for potential external anchors

Anchor	Change level	*n*	Mean (SD) change on AQoLS total	Correlation (95% CI) with change in AQoLS total
PGI-S change between baseline and 3 months	Improved 3 or 4 points	10	− 37.4 (23.0)	0.42 (0.26, 0.56)
	Improved 2 points	17	− 24.7 (22.9)	
	Improved 1 point	29	− 18.2 (16.8)	
	No change	46	− 2.5 (17.6)	
	Worsened 1 to 3 points	20	− 10.8 (14.5)	
PGI-C score at 3 months	Very much better	36	− 18.7 (20.6)	0.28 (0.11, 0.44)
	Much better	34	− 17.6 (17.9)	
	A little better	29	− 13.2 (26.8)	
	No change	18	− 3.5 (7.9)	
	A little or much worse	6	4.2 (18.9)	
CGI-S change between baseline and 3 months	Improved 3 to 5 points	10	− 24.3 (23.9)	0.22 (0.05, 0.38)
	Improved 2 points	15	− 15.1 (19.6)	
	Improved 1 point	23	− 19.1 (19.7)	
	No change	64	− 12.2 (20.9)	
	Worsened 1 to 3 points	12	− 1.0 (16.8)	
CGI-I score at 3 months	Very much improved	25	− 16.8 (18.4)	0.20 (0.03, 0.37)
	Much improved	26	− 16.6 (21.9)	
	Minimally improved	24	− 19.2 (20.9)	
	No change	36	− 9.8 (21.5)	
	Minimally worse to very much worse	13	− 2.8 (18.3)	
DRL change between baseline and 3 months	Decreased 2 or 3 levels	16	− 32.1 (21.0)	0.34 (0.17, 0.48)
	Decreased 1 level	15	− 13.5 (17.1)	
	No change	89	− 11.1 (19.6)	
	Increased 1 to 3 levels	4	− 0.5 (27.0)	
DRL response	Response	54	− 21.5 (19.8)	0.34 (0.17, 0.48)
	Non-response (change that did not meet the criteria for DRL response)	30	− 8.2 (20.5)	

DRL response was defined as change in DRL between baseline and 3 months: from very high to medium or below, from high or medium to low or below, or from low to no alcohol consumption

Moderate improvements (defined as PGI-S improvement of 2, PGI-C score of 2, or a DRL response) corresponded to mean changes in AQoLS-Japan total scores of − 24.7 based on PGI-S, − 17.6 based on PGI-C, and − 21.5 based on DRL. Based on the sensitivity and specificity of ROC in determining a responder definition, an appropriate responder definition on the AQoLS-Japan total could be considered as an improvement of between 8.8 and 14.7 points. Cumulative distribution function plots showed that 50% of patients who had a PGI-C score of 2 (much better) achieved an improvement of between 15 and 20 points on the AQoLS-Japan total. For change in DRL, 50% of patients whose drinking decreased by one DRL achieved an improvement of 10 and 15 points on the AQoLS-Japan total, whereas 50% of patients whose drinking decreased by two or three DRLs achieved an improvement of approximately 30 points on the AQoLS-Japan total.

## Discussion

The primary aim of this study was to evaluate the recently translated AQoLS-Japan. We show that the AQoLS-Japan is a reliable and valid measure of HR-QoL that is able to demonstrate benefits associated with treatment of patients with AUD.

Although the results suggested that the individual dimensions are consistent and that the dimensional structure holds, a number of dimensions in the Japanese version had Cronbach’s alpha coefficients above the recommended optimal range (0.70–0.90), suggesting redundancy among the items of the AQoLS-Japan and the model fit was not as strong as for the UK and French versions. For example, inter-item correlations showed potential redundancy for the items of activities and control. Such findings confirm those of the previous validation study of the measure in the UK and France, which also highlighted the potential to reduce the number of items and/or to simplify the dimensional structure [11]. Although any attempts to remove items would need to be carefully considered in relation to the face and content validity of the overall measure, it is considered that simplification of the AQoLS-Japan could provide a more practical and efficient assessment of HR-QoL in the context of clinical trials and routine clinical practice.

Our results demonstrate good support for the construct validity of the Japanese AQoLS. Correlations with the SF-36, EQ-5D, as well as the patient- and clinician-reported global assessments were as expected, and the pattern of correlations was similar to the pattern obtained with the UK and French versions of the AQoLS [11]. As with the earlier validation study (English/French versions) [11], AQoLS-Japan total scores were more strongly associated with the SF-36 mental component scores than the physical component scores. Thus, our data support the wider literature showing that alcohol dependence impacts mental health more than the physical components of HR-QoL [26, 27]. In our study, we also sought to understand known-groups validity of the scale (not previously reported for the English and French versions). The AQoLS-Japan was consistently able to discriminate between groups based on patient- and clinician-reported severity of disease (PGI-S and CGI-S) and consumption measures (drinking days, HDDs, and DRL). Overall, the total, activities, and control dimensions of the AQoLS had the greatest discriminating ability across levels of consumption. The least discriminatory AQoLS dimensions were relationships and living conditions, which may reflect the Japanese tolerance to excessive alcohol consumption behavior [5, 6].

A limitation of this study is that we did not test whether the AQoLS-Japan is more sensitive than generic measures of HR-QoL (SF-36 and EQ-5D). However, we demonstrated the strength of responsiveness among patients with defined improvements in the global impression assessments and alcohol consumption measures and this was shown by the moderate to strong effect-size estimates for change associated with defined improvements in the global impression assessments and alcohol consumption measures. Ascertaining the MCID can help assess whether a statistically significant treatment effect in a clinical trial is sufficiently large enough to be interpreted as clinically significant and could help personalize treatment in clinical practice. Overall, for the AQoLS-Japan total score, a MCID of between 10 and 15 points is recommended for interpreting group-level differences or change and a responder definition of between 15 and 25 points is recommended for evaluating individual-level change. It is currently recommended that estimation of MCID for any given scale should be based on multiple approaches and triangulation of methods [23]. In our analyses, the MCID estimate for a group-level difference arising from the DRL anchor was considerably lower than the other estimates (PGI-C and PGI-S). This may be because each DRL includes a broad range of consumption, making it possible for patients to have a meaningful change in HR-QoL without an associated change in risk level. Thus, the mean score difference between a reduction of one DRL and no change (i.e., 2.4 points) may be an underestimate of the true MCID for the AQoLS-Japan.

Strengths of the study include its prospective, longitudinal design and the high number of treated patients; the study was focused on clinical practice and included physicians from across Japan. However, this naturalistic design was reflected in the relatively high percentage of patients with low to medium DRL and we may have seen less redundancy of items if we had included more patients with more severe alcohol dependence. The inclusion of low risk DRL also restricted our ability to assess response by reductions in DRL (since, by definition, response in patients at low DRL could be assessed only when they reached zero alcohol consumption after treatment). In addition, most patients had abstinence as their treatment goal, whereas there is a move in the field towards reduced drinking as an alternative goal that is often more acceptable to patients [6, 28]. Such limitations should be considered when using our data or the AQoLS-Japan for designing a clinical trial where the focus of treatment may be reduction of alcohol (vs. abstinence) in patients at higher DRL.

## Conclusion

This psychometric evaluation indicates that the AQoLS-Japan is a reliable and valid measure of HR-QoL that can demonstrate benefits associated with treatment. Our data indicate a shorter version could be developed, which may make it easier to use in clinical research and practice. Our comprehensive approach to characterizing the scale psychometrics will aid future clinical trial design in Japanese subjects with AUD, who carry significant health burdens.

## Electronic supplementary material

Below is the link to the electronic supplementary material.

Supplementary material 1 (PDF 261 kb)
