# Supplementary material for: Psychometric validation of the Japanese version of Alcohol Quality of Life Scale (AQoLS-Japan) in the treatment of patients with alcohol use disorder
Source: Qual Life Res. 2019 Oct 4;29(1):223–35. doi: 10.1007/s11136-019-02310-w (PMC6962253; doi:10.1007/s11136-019-02310-w)
Supplement: Supplementary file 1 — Supplementary material 1 (PDF 261 kb) [file 11136_2019_2310_MOESM1_ESM.pdf]

Psychometric validation of the Japanese version of Alcohol Quality of Life Scale (AQoLS-Japan) in the treatment of patients with alcohol use disorder

Susumu Higuchi MD, PhD, Yoshiya Moriguchi MD, PhD, Kristin Hui Xian Tan, MPH

Corresponding author: Professor Susumu Higuchi, Director, National Hospital Organization Kurihama Medical and Addiction Center, 5-3-1 Nobi, Yokosuka, Kanagawa, 239-0841, Japan

h-susumu@db3.so-net.ne.jp

Table e1. AQoLS UK Version

Over the last 4 weeks this has applied to me ...Not at all/A little/Quite a lot/Very much

1	I have felt I miss out on everyday activities with family and friends
2	It has been difficult for me to follow through on plans
3	I have been restricted in the places I can go because of alcohol
4	I have struggled doing physical activities due to alcohol (e.g. DIY, walking, cycling)
5	It has been too much effort to do jobs around the house due to alcohol (e.g. cleaning, gardening, maintaining the house)
6	Alcohol has interfered with my ability to work
7	I have cut myself off from other people
8	I have neglected the people close to me
9	Alcohol has damaged my close relationships
10	I have behaved badly towards other people
11	I have felt I miss out on family life because of alcohol
12	I have felt that people have no trust in me
13	Alcohol has interfered with my sex life
14	Alcohol has interfered with my relationships with friends
15	I have struggled to keep on top of my everyday household affairs (e.g. dealing with mail, organizing appointments)
16	Alcohol has had a negative effect on my housing situation
17	All my money has gone on alcohol
18	Alcohol has caused me financial difficulties
19	I have felt ashamed of myself
20	I have felt that people look down on me
21	I have felt that I am wasting my life
22	I have worried about the effect alcohol has been having on my health
23	I have worried about alcohol causing problems in my life
24	I have had no appetite
25	I have neglected my appearance
26	I have neglected my general health
27	I have put myself in risky situations
28	I have felt that nothing matters more than alcohol
29	Alcohol has controlled me
30	My life has revolved around alcohol
31	I have planned my days around alcohol
32	I have felt as though I have not been in control of myself
33	I have not had a good night's sleep
34	I have not been getting enough sleep

Table e2 Baseline characteristics by Drinking Risk Level (DRL)

	Low or medium DRL (N=117)	High or very high DRL (N=33)
Sex; n (%)		
Male	97 (82.9%)	26 (78.8%)
Female	20 (17.1%)	7 (21.2%)
Age (years); mean (SD)	53.3 (12.7)	51.3 (10.0)
Living status; n (%)		
Alone	44 (37.6%)	14 (42.4%)
Not alone	73 (62.4%)	19 (57.6%)
Employment status; n (%)		
On a job	44 (37.6%)	18 (54.5%)
Working at home	3 (2.6%)	3 (9.1%)
Retired	14 (12.0%)	4 (12.1%)
No job	56 (47.9%)	8 (24.2%)
Current smoker; n (%)	80 (69.0%)	20 (60.6%)
Alcohol use disorder; n (%)		
Abuse	4 (3.4%)	0 (0.0%)
Dependence	113 (96.6%)	33 (100.0%)
Total alcohol consumption (g/day) over 28 days; mean (SD)	15.8 (18.6)	112.5 (44.3)
Number of drinking days; mean (SD)	8.4 (10.0)	23.8 (5.0)
Number of heavy drinking days; mean (SD)	4.4 (7.4)	23.1 (5.4)
Age at onset of drinking problem (years); mean (SD)	38.4 (13.9)	41.2 (12.6)
Time since diagnosis (years); mean (SD)	4.2 (5.4)	2.3 (5.1)
Number of previous attempts to abstain or reduce alcohol consumption; mean (SD)	9.9 (21.9)	4.7 (5.9)
Comorbidities; n (%)		
Cardiovascular	26 (22.2%)	7 (21.2%)
Gastric	24 (20.5%)	4 (12.1%)
Hepatic	49 (41.9%)	15 (45.5%)
Metabolic	23 (19.7%)	4 (12.1%)
Psychiatric	44 (37.6%)	11 (33.3%)

Table e3 Generic quality of life measures at Baseline and follow-up visits

		Baseline (n=150)	Week 2 (n=145)	Month 3 (n=133)
**EQ-5D-3L Mobility**	I have no problems in walking about<br>I have some problems in walking about<br>I am confined to bed<br>Missing (n)	118 (80.3%)<br>26 (17.7%)<br>3 (2.0%)<br>3	123 (86.6%)<br>17 (12.0%)<br>2 (1.4%)<br>3	110 (85.9%)<br>18 (14.1%)<br>0 (0.0%)<br>5
**EQ-5D-3L Self-care**	I have no problems with self-care<br>I have some problems washing/dressing myself<br>I am unable to wash or dress myself<br>Missing (n)	129 (87.2%)<br>19 (12.8%)<br>0 (0.0%)<br>2	134 (93.1%)<br>10 (6.9%)<br>0 (0.0%)<br>1	124 (95.4%)<br>5 (3.8%)<br>1 (0.8%)<br>3
**EQ-5D-3L Usual activities**	I have no problems performing my usual activities<br>I have some problems performing my usual activities<br>I am unable to perform my usual activities<br>Missing (n)	110 (74.3%)<br>31 (20.9%)<br>7 (4.7%)<br>2	114 (79.7%)<br>25 (17.5%)<br>4 (2.8%)<br>2	111 (84.1%)<br>19 (14.4%)<br>2 (1.5%)<br>1
**EQ-5D-3L Pain/discomfort**	I have no pain or discomfort<br>I have moderate pain or discomfort<br>I have extreme pain or discomfort<br>Missing (n)	91 (62.3%)<br>48 (32.9%)<br>7 (4.8%)<br>4	86 (59.7%)<br>50 (34.7%)<br>8 (5.6%)<br>1	87 (66.9%)<br>36 (27.7%)<br>7 (5.4%)<br>3
**EQ-5D-3L Anxiety/depression**	I am not anxious or depressed<br>I am moderately anxious or depressed<br>I am extremely anxious or depressed<br>Missing (n)	84 (57.1%)<br>53 (36.1%)<br>10 (6.8%)<br>3	88 (61.1%)<br>48 (33.3%)<br>8 (5.6%)<br>1	94 (71.8%)<br>34 (26.0%)<br>3 (2.3%)<br>2
**Score in VAS**	Mean (SD)<br>Median (Q1-Q3)<br>Range<br>Missing (n)	68.5 (20.3)<br>70.0 [60.0-81.5]<br>(8.0, 100.0)<br>9	70.4 (21.0)<br>75.0 [60.0-82.0]<br>(7.0, 100.0)<br>11	75.3 (18.7)<br>80.0 [70.0-90.0]<br>(10.0, 100.0)<br>6
**EQ-5D-3L index value**	Mean (SD)<br>Median (Q1-Q3)<br>Range<br>Missing (n)	0.8 (0.2)<br>0.8 [0.7-1.0]<br>(0.1, 1.0)<br>5	0.8 (0.2)<br>0.8 [0.7-1.0]<br>(0.2, 1.0)<br>4	0.8 (0.2)<br>0.8 [0.7-1.0]<br>(0.2, 1.0)<br>7
**SF-36: Physical functioning**	Mean (SD)<br>Median (Q1-Q3)<br>Range<br>Missing (n)	85.7 (16.3)<br>90.0 [80.0-95.0]<br>(10.0, 100.0)<br>0	84.4 (18.2)<br>90.0 [75.0-95.0]<br>(15.0, 100.0)<br>0	86.6 (16.0)<br>90.0 [80.0-100.0]<br>(35.0, 100.0)<br>3
**SF-36: Role limitation due to physical health**	Mean (SD)<br>Median (Q1-Q3)<br>Range<br>Missing (n)	73.3 (26.3)<br>75.0 [56.3-100.0]<br>(0.0, 100.0)<br>0	73.0 (25.4)<br>75.0 [50.0-100.0]<br>(0.0, 100.0)<br>0	77.3 (24.5)<br>87.5 [58.3-100.0]<br>(0.0, 100.0)<br>3
**SF-36: Role limitation due to emotional problems**	Mean (SD)<br>Median (Q1-Q3)<br>Range<br>Missing (n)	72.0 (26.6)<br>75.0 [58.3-100.0]<br>(0.0, 100.0)<br>0	71.7 (28.1)<br>75.0 [50.0-100.0]<br>(0.0, 100.0)<br>0	78.1 (25.6)<br>91.7 [58.3-100.0]<br>(0.0, 100.0)<br>3
**SF-36: Vitality**	Mean (SD)<br>Median (Q1-Q3)<br>Range<br>Missing (n)	58.4 (20.8)<br>56.3 [50.0-75.0]<br>(0.0, 100.0)<br>1	57.2 (24.0)<br>56.3 [43.8-75.0]<br>(0.0, 100.0)<br>0	64.1 (20.3)<br>68.8 [50.0-81.3]<br>(0.0, 100.0)<br>2
**SF-36: Mental health**	Mean (SD)<br>Median (Q1-Q3)<br>Range<br>Missing (n)	63.0 (21.7)<br>65.0 [50.0-80.0]<br>(5.0, 100.0)<br>1	62.6 (22.2)<br>70.0 [45.0-80.0]<br>(0.0, 100.0)<br>0	68.1 (21.3)<br>70.0 [55.0-85.0]<br>(10.0, 100.0)<br>2
**SF-36: Social functioning**	Mean (SD)<br>Median (Q1-Q3)<br>Range<br>Missing (n)	74.5 (27.2)<br>75.0 [50.0-100.0]<br>(0.0, 100.0)<br>1	73.7 (26.4)<br>75.0 [50.0-100.0]<br>(12.5, 100.0)<br>0	78.5 (26.0)<br>87.5 [62.5-100.0]<br>(0.0, 100.0)<br>1

**SF-36: Pain**	Mean (SD)<br>Median (Q1-Q3)<br>Range<br>Missing (n)	68.2 (25.1)<br>72.0 [46.5-92.0]<br>(0.0, 100.0)<br>1	67.7 (25.7)<br>72.0 [52.0-84.0]<br>(0.0, 100.0)<br>0	68.8 (26.4)<br>72.0 [52.0-100.0]<br>(10.0, 100.0)<br>2
**SF-36: General health**	Mean (SD)<br>Median (Q1-Q3)<br>Range<br>Missing (n)	55.1 (17.4)<br>56.0 [45.0-67.0]<br>(10.0, 100.0)<br>1	54.6 (18.5)<br>57.0 [45.0-67.0]<br>(0.0, 100.0)<br>1	58.0 (20.2)<br>57.0 [47.0-72.0]<br>(0.0, 100.0)<br>3

Table e4. Confirmatory factor analysis: AQoLS interfactor correlations (SE) at Baseline and Week 2

**AQoLS Dimension**		Activities	Relationships	Living Conditions	Negative Emotions	Control	Sleep	Self-esteem
Activities	Baseline	1.00 (-)						
Relationships	Baseline	0.90 (0.02)	1.00 (-)					
Living Conditions	Baseline	0.80 (0.04)	0.86 (0.04)	1.00 (-)				
Negative Emotions	Baseline	0.84 (0.03)	0.79 (0.04)	0.68 (0.06)	1.00 (-)			
Control	Baseline	0.88 (0.02)	0.84 (0.03)	0.76 (0.05)	0.78 (0.05)	1.00 (-)		
Sleep	Baseline	0.53 (0.06)	0.52 (0.07)	0.51 (0.06)	0.52 (0.08)	0.52 (0.07)	1.00 (-)	
Self-esteem	Baseline	0.84 (0.03)	0.97 (0.02)	0.79 (0.04)	0.85 (0.04)	0.80 (0.04)	0.42 (0.07)	1.00 (-)

アルコール
クオリティ・オブ・ライフ スケール
(AQoLS)

<u>指示をよく読んでください</u>

本アンケートの目的は、**<u>過去4週間に</u>**、アルコールが及ぼした影響の程度を調べることです。過去**<u>4週間</u>**より前のことは回答しないでください。

ここでいう「アルコール」とは、過去4週間におけるアルコールとの全般的な関わりであって、その間飲酒していたかどうかは問いません。

<u>各</u>質問に対し、最も当てはまる回答を選んでください。

過去4週間に当てはまる回答...

	全くない	少し	かなり	非常に
1. 家族や友人との日常的な活動の機会を逸していると感じていた	❑	❑	❑	❑
2. 予定されていた計画を、最後までやり通すのは困難であった	❑	❑	❑	❑
3. アルコールのせいで、出かけられる場所が限られていた	❑	❑	❑	❑
4. アルコールのせいで、身体的な活動が困難であった（例：日曜大工、散歩、運動）	❑	❑	❑	❑
5. アルコールのせいで、家の周りの事をするのに大変な努力を要していた（例：掃除、ガーデニング、家のメンテナンス）	❑	❑	❑	❑
6. アルコールのせいで、仕事をする能力に支障が出ていた	❑	❑	❑	❑

裏返してください

© Copyright 2013 HJ Aubin, A Luquiens & S Higuchi

過去4週間についてのみ回答してください。

	過去4週間に当てはまる回答...			
	全くない	少し	かなり	非常に
7. 他の人との繋がりを断ち切っていた	❑	❑	❑	❑
8. 親しい人をおろそかにしていた	❑	❑	❑	❑
9. アルコールのせいで、身内との人間関係が損なわれていた	❑	❑	❑	❑
10. 他の人に対して、ひどい態度を取ってしまっていた	❑	❑	❑	❑

	過去4週間に当てはまる回答...			
	全くない	少し	かなり	非常に
11. アルコールのせいで、家庭生活の機会を逸していると感じていた	❑	❑	❑	❑
12. 人に信用されていないと感じていた	❑	❑	❑	❑
13. アルコールのせいで、性生活に支障が出ていた	❑	❑	❑	❑
14. アルコールのせいで、友人との関係に支障が出ていた	❑	❑	❑	❑

裏返してください

© Copyright 2013 HJ Aubin, A Luquiens & S Higuchi

過去4週間についてのみ回答してください。

過去4週間に当てはまる回答...	全くない	少し	かなり	非常に
15. 日常の家事を適切にこなし続けることが困難であった（例：郵便物の処理、請求への支払）	❑	❑	❑	❑
16. アルコールのせいで、住宅事情に悪影響があった（例：立ち退き、実家に転居）	❑	❑	❑	❑
17. 自分のお金を全て、アルコールに使ってしまった	❑	❑	❑	❑
18. アルコールのせいで、財政難に陥った	❑	❑	❑	❑

過去4週間に当てはまる回答...	全くない	少し	かなり	非常に
19. 自分のことを恥ずかしく感じていた	❑	❑	❑	❑
20. 人に見下されていると感じていた	❑	❑	❑	❑
21. 人生を無駄にしていると感じていた	❑	❑	❑	❑
22. アルコールによる健康への影響が心配であった	❑	❑	❑	❑
23. 生活に問題を引き起こすアルコールのことが心配であった	❑	❑	❑	❑

裏返してください

© Copyright 2013 HJ Aubin, A Luquiens & S Higuchi

<u>過去4週間についてのみ</u>回答してください。

	全くない	少し	かなり	非常に
	過去4週間に当てはまる回答...			
24. 食欲が無かった	❑	❑	❑	❑
25. 外見をおろそかにしていた	❑	❑	❑	❑
26. 自分の健康全般をおろそかにしていた	❑	❑	❑	❑
27. 危険な状況に身を置いていた（例：事故、けんか、法的トラブルに巻き込まれる）	❑	❑	❑	❑

	全くない	少し	かなり	非常に
	過去4週間に当てはまる回答...			
28. アルコールよりも大事なことはないと感じていた	❑	❑	❑	❑
29. アルコールに支配されていた	❑	❑	❑	❑
30. 生活はアルコール中心に回っていた	❑	❑	❑	❑
31. アルコール中心に日々の計画を立ててきた	❑	❑	❑	❑
32. 自分をコントロールできていないと感じていた	❑	❑	❑	❑

裏返してください

© Copyright 2013 HJ Aubin, A Luquiens & S Higuchi

過去4週間についてのみ回答してください。

	過去4週間に当てはまる回答...			
	全くない	少し	かなり	非常に
33. 夜良く眠ることができていなかった	❑	❑	❑	❑
34. 十分な時間睡眠が取れていなかった	❑	❑	❑	❑

アンケートにご協力いただきありがとうございます。

最初に戻って、各ページのすべての質問に回答していることを確認してください。

© Copyright 2013 HJ Aubin, A Luquiens & S Higuchi
